# Occurrence, Antimicrobial Resistance and Molecular Diversity of *Enterococcus* *faecium* in Processed Pork Meat Products in Korea

**DOI:** 10.3390/foods9091283

**Published:** 2020-09-12

**Authors:** Hyun Jung Kim, Minseon Koo

**Affiliations:** 1 Research Group of Consumer Safety, Korea Food Research Institute, Wanju, Jeollabuk-do 55365, Korea; 2 Department of Food Biotechnology, University of Science and Technology, Daejeon 34113, Korea; minsk@kfri.re.kr; 3 Food Analysis Center, Korea Food Research Institute, Wanju, Jeollabuk-do 55365, Korea

**Keywords:** *Enterococcus faecium*, processed pork meat products, antimicrobial resistance, rep-PCR, molecular typing

## Abstract

Because *Enterococcus faecium* is an important nosocomial pathogen and sentinel organism for tracking antimicrobial resistance, information on the contamination and antimicrobial resistance patterns of *E. faecium* in food are essential to public health and food safety. We analyzed the occurrence of *E. faecium* in retail pork meat products (*n* = 124), and antimicrobial resistance of 30 *E. faecium* isolates were examined against 14 antimicrobials using the broth dilution test and disc diffusion test. Rep-PCR-based molecular diversity was also analyzed using Deviersilab. The highest contamination of enterococci was observed for minced pork meat but most of the *E. faecium* was isolated from meatball-type frozen pork meat products (FP). Incidences of antimicrobial-resistant *E. faecium* against erythromycin, clindamycin and nitrofurantoin were 80%, 50% and 20%, respectively. No vancomycin-resistant enterococci were analyzed. Rep-PCR showed distinctive clusters with a similarity ≥ 98%, consisting of 18 *E. faecium* isolates from FP manufactured in seven companies. The analyzed data on the contamination and antimicrobial resistance patterns combined with molecular typing can be useful to derive risk management of antimicrobial-resistant enterococci in food.

## 1. Introduction

Enterococci are gram-positive, facultative anaerobic bacteria that are frequently detected in the intestinal tracts of animals and humans as a part of the normal microbiota [1]. Generally they are not harmful to humans, but some species of *Enterococcus* are known as nosocomial agents in hospitalized and immunocompromised patients in hematology, oncology, and transplantation surgery [1]. Enterococci are a major cause of sepsis worldwide and are among the leading nosocomial pathogens and account for about 10% of hospital-acquired bacteremia cases [2]. Antimicrobial resistance is known to contribute to increased morbidity and mortality in infections caused by Enterococcus [3]. They can be transmitted not only person-to-person, but also through contaminated environments including foods, causing a variety of infections, such as bacteremia, endocarditis, and urinary tract infections [4,5,6,7].

As enterococci are part of the normal microbiota of animals and they are able to survive in biotic and abiotic environments, they are also found in foods of animal and plant origin. Enterococci are recognized as a hygienic indicator in foods and as sentinel organisms for tracking antimicrobial resistance trends [1,8]. In the food industry, some strains of enterococci have been used as probiotics for human and animal use, as well as starter cultures for fermented food production [5,9]. Enterococci play a beneficial role in the production of traditional fermented foods, giving unique organoleptic properties [5,10]. However, their ability to harbor and easily acquire virulence and antibiotic resistance genes through horizontal gene transfer hampers their use as probiotics or as starter/adjunct cultures in foods [11].

The most frequently detected enterococcus species in clinical and food samples of greatest importance to human health are *Enterococcus faecalis* and *Enterococcus faecium.* Both species cause a variety of infections in immunocompromised patients [7]. They show resistance to antimicrobials including β-lactams, high-levels of aminoglycosides, and glycopeptides [6]. Formerly, 90% of the human infections caused by enterococci were due to *E. faecalis*, and the remaining 10% were known to be related to *E. faecium* [11,12]. However, recent data have shown that much more frequent incidences of antimicrobial resistance against vancomycin, ampicillin, and high levels of aminoglycosides can be observed in *E. faecium* compared to *E. faecalis* [11,13]. Antimicrobial-resistant *E. faecium* as a conditional pathogen with low pathogenicity affects above all immunocompromised patients and possibly cause systemic infections limiting the choice of an effective antibiotics.

Foodborne transmission of enterococci may affect a larger part of the population through consumption and handling of contaminated poultry meat and other food items that may be cross-contaminated in the kitchen [14]. Despite the availability of anti-gram-positive agents (e.g., linezolid, quinupristin/dalfopristin, daptomycin, tigecycline), enterococci have rapidly adapted and resistance has emerged to all these newer agents [15]. Because foods are important vehicles for transmitting antimicrobial-resistant enterococci, contamination and antimicrobial resistance patterns of enterococci in food are essential to public health and food safety with respect to horizontal transfer of genes conferring resistance to antibiotics that are relevant for treatment of enterococcal infections.

However, most studies on the prevalence of antimicrobial-resistant enterococci have focused on *E. faecalis* in Korea. Processed pork meat products are popular food dishes that are frequently provided in restaurants and school canteen services in Korea [16], possible contamination of antimicrobial-resistant *E. faecium* in pork meat dishes can cause a food safety and public health burden. However, information on the antimicrobial resistance of *E. faecium*, especially in retail pork meat products, is limited [17]. In this study, the occurrence of *E. faecium* in retail pork meat products and the antimicrobial resistance of the isolates were examined. Automated repetitive-sequence-based PCR (rep-PCR) was used to investigate the strain relatedness of *E. faecium* isolates obtained from pork meat products and to provide molecular epidemiology data useful for risk management of enterococci with particular antibiotic resistance traits.

## 2. Materials and Methods

### 2.1. Sample Collection and Preparation

Samples of processed pork meat products were collected from eight retail markets located in Gyonggi Province in South Korea. The 124 samples included minced pork meat (MP, *n* = 40), ready-to-cook pork meat products seasoned with fermented red pepper paste (SRP, n = 34) or seasoned with soy sauce (SSP, *n* = 34), and meatball-type frozen processed pork meat products (FP, *n* = 16). Samples were placed on ice in a cooling box after purchase and were transported to the laboratory within 2 h. Samples that included MP, SRP and SSP were immediately stored at 4 °C. FP samples were kept at −20 °C until analysis.

### 2.2. Isolation and Identification of Enterococcus spp.

The enumeration of enterococci was carried out according to ISO-7899-1 [9,18] with some modifications. In brief, 25 g of the sample was cut into small pieces with sterilized scissors and homogenized with 225 mL sterile peptone water in a Stomacher^®^ 400 Circulator (Seward, Worthing, UK) for 2 min at 230 rpm. The homogenates were subjected to serial 10-fold dilution with peptone water, and then 100 μL of each dilution was spread on Bile Aesculin Azide (BAAA) agar (MERCK, Darmstadt, Germany) plates. After incubation at 37 °C for 24 h, the plates with typical numbers of colonies between 25 and 250 were selected for presumptive enumeration of *Enterococcus* spp. At least five presumptive enterococci colonies with typical color on BAAA agar were selected and transferred to Tryptic soy agar (TSA, Merck, Darmstadt, Germany) and were identified using biochemical testing in a VITEK^®^ 2 compact system (bioMérieux, Marcy I’Etoile, France).

### 2.3. Antimicrobial Resistance

Antimicrobial resistance of the *E. faecium* isolates was tested by the broth dilution method and disc diffusion method. The broth dilution test was carried out using an AST-P601 test card in a VITEK^®^ 2 compact system (bioMérieux, Marcy I’Etoile, France) according to the manufacturer’s instructions, and the disc diffusion test was performed with the antibiotic susceptibility testing disc (Oxoid Ltd., Basingstoke, UK) based on the Clinical and Laboratory Standards Institute (CLSI) standards [19]. The tested antimicrobials were as follows: ciprofloxacin, erythromycin, linezolid, teicoplanin, vancomycin, tetracycline, tigecycline, nitrofurantoin, ampicillin, amoxycillin/clavulanic acid, chloramphenicol, quinupristin/dalfopristin, clindamycin and gentamicin. The qualitative interpretation (resistant or sensitive) is based on the breakpoints for enterococci provided by the CLSI standards [19]. *E. faecalis* ATCC 29212 was used as a control strain to assess the quality of antimicrobial resistance testing, and all values were within the accepted limits [19].

### 2.4. Rep-PCR-Based Molecular Typing

An automated repetitive-sequence-based PCR (rep-PCR) system (DiversiLab, bioMérieux, Marcy I’Etoile, France) was used to analyze the genetic similarities of the *E. faecium* strains. DNA of the *E. faecium* was extracted using the UltraClean microbial DNA isolation Kit (Mo Bio Laboratories, Solona Beach, CA, USA) and was standardized to a concentration of ca. 25 ng/μL. For the rep-PCR amplification of non-coding intergenic repetitive elements in the genomic DNA, the DiversiLab *Enterococcus* Kit (Bacterial Barcodes, Inc., Athens, GA, USA) was used according to the manufacturer’s instructions. PCR conditions were as follows: initial denaturation at 94 °C for 5 min; 30 cycles at 94 °C for 1 min, 56 °C for 1 min and 72 °C for 1 min; with the final extension at 72 °C for 10 min. Amplicons were analyzed using the DiversiLab system, which includes fragment separation using microfluidic chips and Agilent B2100 Bioanalyzer (Agilent Technologies Inc., Santa Clara, CA, USA). The DNA standard markers for the normalization of sample runs, and Chip kit molecular weight ladders were used. Results were analyzed using the DiversiLab software (version 3.3). 

## 3. Results and Discussion

### 3.1. Contamination of Enterococcus spp.

**Figure 1:**
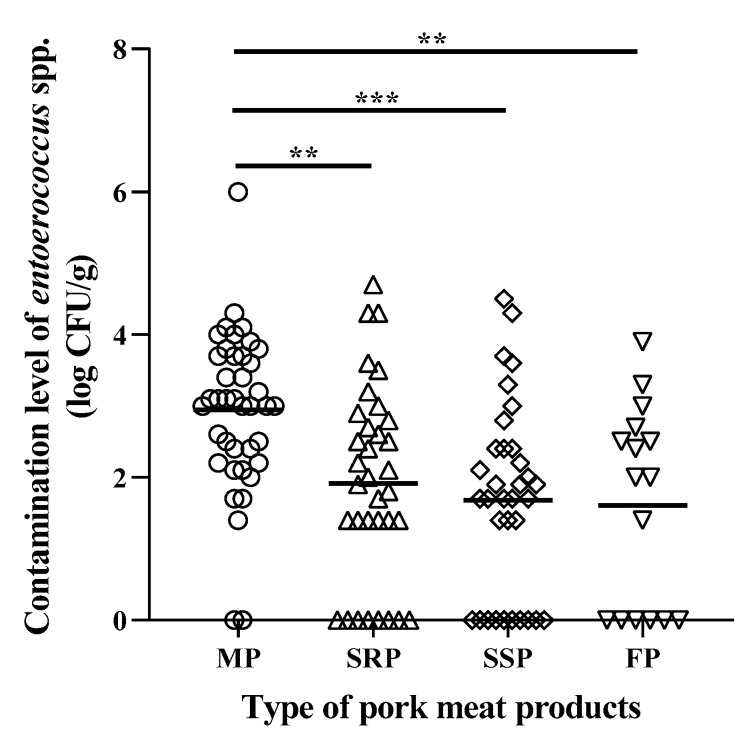
Contamination level of *Enterococcus* spp. in commercial processed pork meat products. Types of pork meat products include minced pork meat (MP), ready-to-cook pork meat products seasoned with red pepper paste (SRP), ready-to-cook pork meat products seasoned with soy sauce (SSP) and meatball-type frozen processed pork meat products (FP). Horizontal bars indicate the mean of the contamination level of enterococci of each sample. Tukey’s multiple comparisons test was performed to identify the statistical significance of the contamination level of *Enterococcus* spp. in different types of pork meat products. **: *p* < 0.01, ***: *p* < 0.001.

Contamination levels of *Enterococcus* spp. in different types of processed pork meat products are presented in Figure 1. The mean contamination level of *Enterococcus* spp. was 3.0, 1.9, 1.7 and 1.6 log cfu/g for MP, SRP, SSP and FP, respectively. The highest contamination of *Enterococcus* spp. was observed in MP (3.0 log cfu/g) with statistical significance compared with other types of processed pork meat products (*p* < 0.01). The contamination level of *Enterococcus* spp. in MP was obviously higher than SSP samples with statistical significance (*p* < 0.001). There are no statistically significant differences between SRP, SSP and FP.

The mean load of *Enterococcus* spp. observed in MP was similar to the contamination in conventional chicken carcasses (2.9 ± 0.4 log CFU/mL). In the same study, the contamination level of enterococci in organic chicken carcasses was 1.8 ± 0.3 log CFU/mL [20], which is similar to the contamination level of enterococci in SRP, SSP and FP in this study. A total of 30 *E. faecium* isolates were obtained from the processed pork meat products, which comprised 2 isolates from MP, 6 isolates from SRP, 2 isolates from SSP and 20 isolates from FP.

Prevalence of *E. faecalis* and *E. faecium* in the pork meat samples suggested different patterns of contamination, i.e., *E. faecium* was the most frequently isolated from FP followed by SRP, MP and SSP, while the highest prevalence of *E. faecalis* was observed in FP as well as MP, as shown in Table 1. Interestingly, the prevalence of *E. faecium* in the total sample was 15.3% (Table 1), but *E. faecium* was isolated from 68.8% of the FP samples, which suggested that some of the common ingredients might be contaminated with *E. faecium*, or *E. faecium* might be cross-contaminated from processing environments, including water.

Other enterococci, including *E. avium*, *E. raffinose*, *E. casseliflavus*, *E. gallinarum*, *E. durans*, were also isolated from processed pork meat products, which is supported by the previous study on the isolation of enterococci, i.e., *Enterococcus* spp., including *E. hirae*, *E. casselifavus*, *E. durans*, *E. devriesei*, *E. gilvus*, *E. mundtii*, and *E. thailandicus*, which were also isolated from beef and pork samples [11]. SRP and SSP contained a variety of ingredients including seasoning and fresh vegetables such as garlic, green onion and onions, and thus, different enterococci might originate from seasoning and raw vegetables. FP also contains different types of seasoning and vegetables and even chicken or beef as ingredients, but relatively less diverse enterococci, including *E. casseliflavus*, *E. faecalis* and *E. faecium* were isolated.

In this study, *E. faecium* and *E. faecalis* were observed in 15.3% and 20.2% of the processed pork meat products, while a previous study suggested that *E. faecium* and *E. faecalis* was detected in 11.3% and 69.5% of red meat [11]. In Argentinean artisanal dry fermented sausages, the most frequently isolated enterococci was *E. faecium* (56%), followed by *E. faecalis* (17%) and other species (*E. durans*, *E. casseliflavus*, and *E. mundtii*) [21].

### 3.2. Antimicrobial Resistance

The antimicrobial resistance of 30 *E. faecium* isolates were analyzed against fourteen antimicrobials belonging to 12 different antimicrobial classes (Table 2). As indicated, 9 out of the 14 tested antimicrobials were critically important antimicrobials for human medicine, and the remaining were highly important antimicrobials for human medicine, as categorized by WHO AGISAR [22].

As shown in Table 2, the highest incidence of antimicrobial resistance was observed for erythromycin (80% resistance) followed by clindamycin (50%) and nitrofurantoin (20%) in the case of *E. faecium* isolates. Resistance and intermediate resistance to erythromycin, nitrofurantoin and clindamycin was detected in 96.7%, 86.6% and 76.6%, respectively. Because erythromycin is considered to be a critically important antimicrobial for human use, high antimicrobial resistance against erythromycin poses a public health problem. Nitrofurantoin is used to treat urinary tract infections caused by *Enterococcus* spp., including vancomycin-resistant enterococci (VRE) [1]. The high prevalence of resistant (20%) and intermediate resistant (66.6%) *E. faecium* against nitrofurantoin in pork meat products suggested that special consideration of the prevalence and mitigation of the spread of antimicrobial-resistant *E. faecium* in the food system is needed. All the tested *E. faecium* isolates were susceptible to vancomycin and teicoplanin in this study. No *E. faecium* isolates were resistant or intermediate resistant to quinupristin/dalfopristin in our study, although enterococci are known as intrinsically resistant to quinupristin/dalfopristin [11].

To compare with the antimicrobial resistance patterns of *E. faecium*, information on the antimicrobial resistance of *E. faecalis* against 11 antimicrobials were adopted from our previous report [23]. Resistance against quinupristin/dalfopristin, clindamycin and gentamicin were further analyzed for 36 *E. faecalis* isolates in this study. The highest resistance of *E. faecalis* was observed for clindamycin (88.9%) followed by quinupristin/dalfopristin (69.4%) and tetracycline (58.3%). Obviously, the high incidence of *E. faecalis* isolates resistant to clindamycin and quinupristin/dalfopristin seems related to intrinsically resistant characteristics of enterococci against clindamycin and quinupristin/dalfopristin [1,24]. In the case of *E. faecalis* isolated from processed pork meat products, a higher incidence of resistant isolates was observed for tetracycline (58.3%), quinupristin/dalfopristin (69.4%) and clindamycin (88.9%) compared to *E. faecium*. Both the *E. faecium* and *E. faecalis* strains are not resistant to vancomycin, ciprofloxacin, linezolid, teicoplanin, tigecycline, amoxycillin/clavulanic acid and ampicillin, which are critically important antimicrobials for human use (Table 2).

National surveillance data on the antimicrobial resistance of *E. faecium* isolated from livestock and livestock products in Korea showed that the highest antimicrobial resistance of *E. faecium* (*n* = 197) isolated from pig feces was observed for erythromycin (48.2%) followed by tigecycline (17.3%) and tetracycline (16.2%) [25]. Generally, *E. faecium* isolated from pig feces showed similar or higher antimicrobial resistance than antimicrobial resistances observed in this study, except for erythromycin [25]. The highest resistance of *E. faecium* (*n* = 14) isolated from domestic produced pork meat products was observed for tetracycline (42.9%) followed by quinupristin/dalfopristin (35.7%), erythromycin (14.3%) and clindamycin (14.3%). In case of *E. faecium* (*n* = 9) isolated from imported pork meat products, the most frequently observed antimicrobial resistances were quinupristin/dalfopristin (55.6%) and tetracycline (22.2%) [25]. No VRE were isolated from pig feces and pork meat products in the previous study [25]. According to a report from the Korean global antimicrobial resistance surveillance system (Kor-GLASS) for 2017, resistance to vancomycin (34.0%, 98/288) and teicoplanin (18.8%, 98/288) was frequently observed in *E. faecium* strains isolated from human [26]. Resistance to erythromycin was not provided in Kor-GLASS [26]. However, another report on the antimicrobial resistance of *E. faecium* human isolates (*n* = 21) obtained from the Asian Bacterial Bank of the Asia Pacific Foundation for Infectious Diseases showed that antimicrobial resistance to ampicillin, ciprofloxacin, erythromycin and tetracycline was 100%, 100%, 95.2% and 33.3%, respectively [27].

Similar to this study, no VRE were isolated from chicken carcasses in Korea [20]. However, 8 fecal and 3 produce isolates out of 389 *Enterococcus* spp. isolates resistant to vancomycin and teicoplanin were reported in Korea [28]. The previously reported VRE isolates showed multidrug-resistant properties against ampicillin, penicillin, erythromycin, rifampin, gentamicin, teicoplanin, vancomycin, streptomycin, etc. VRE isolates from fresh produce seemed isolated from humans because the isolates from fresh produce were shown to have common shared characteristics with the isolates from humans, such as antimicrobial resistance, multilocus sequence typing (MLST), and Tn 1546 transposon analysis [28].

Enterococci are intrinsically susceptible to tetracyclines and erythromycin, although acquired resistance to these agents is widespread (except for tigecycline) [24]. We observed 80.0% and 11.1% resistance against erythromycin for *E. faecium* and *E. faecalis* isolated from processed pork meat products, respectively. In the previous report in Korea, 17.3–44.2% of the *E. faecalis* isolates obtained from chicken carcasses showed resistance to erythromycin [20]. We also observed that 10% and 58.3% of the *E. faecium* and *E. faecalis* strains are resistant against tetracycline, which is considered a highly important antimicrobial for human medicine (Table 2). The newer agents linezolid, tedizolid, daptomycin, televancin, and oritavancin are active against enterococci, and the pristinamycin combination quinupristin/dalfopristin is active against *E. faecium* only [24]. In this study, no resistance against linezolid was observed for *E. faecium* and *E. faecalis* except one intermediate resistant *E. faecalis* to linezolid.

Varied uses of antimicrobials as animal medicines might cause the different antimicrobial resistance patterns of pathogens. For example, incidences of resistance against linezolid, tigecycline and vancomycin were less than 1% in any species or meat source, and the low incidences can be explained by these antimicrobials not being used in food animals. In contrast, tetracyclines are the most heavily used antimicrobials in food animals and they revealed the greatest resistance prevalence [1]. In Korea, tetracyclines, followed by penicillins, were the most frequently sold antimicrobials for veterinary use [29]. The highest resistance rate against tetracycline of *E. faecalis* might be related to the amount of antimicrobials sold in Korea. No VRE observed in this study could be explained by the banning of the use of avoparcin in Korea in 1997. The use of animal growth promoters such as avoparcin is reportedly associated with the appearance of VRE [21,25]. However, in the case of *E. faecium*, only 10% of the isolates possess resistance against tetracycline. Similarly, differences in resistance prevalence between *E. faecalis* and *E. faecium* isolated from retail meat in the United States with statistical significance were observed for tetracycline, gentamycin, erythoromycin, nitrofurantoin, ciprofloxacin and quinupristin/dalfopristin [1].

Among 30 tested *E. faecium*, only 4 isolates were susceptible to all 14 antimicrobials examined. Some of the *E. faecium* isolates (23.3%) were resistant to single antimicrobials (Table 3). Multi-drug resistances (MDR) were observed for 13.3% of the tested *E. faecium* isolates. *E. faecium* isolated from SRP showed the highest incidence of MDR followed by isolates from FP. All of the MDR isolates are resistant to clindamycin. Considering that *E. faecium* are naturally resistant to clindamycin, the incidence of MDR *E. faecium* obtained from processed pork meat produces is quite low. Except for clindamycin, the most frequently observed resistance patterns against two antimicrobials were erythromycin–nitrofurantoin followed by erythromycin–tetracycline.

According to the population-weighted consumption and distribution of antimicrobial agents, the most frequently used for humans were penicillins, followed by 2nd generation cephalosporins, macrolides, and fluoroquinolones, which have 4.52, 4.47, 3.32, and 2.75 defined daily dose per 1000 people per day, respectively in Korea [30]. In the present study, there was no observed resistance against penicillins for *E. faecium* and *E. faecalis*; however, the high resistance of *E. faecium* (80%) against erythromycin suggested that risk management for antimicrobial resistance is needed, considering the importance of antimicrobials for human use and possible cross-contamination via food [4,5].

### 3.3. Analysis of Strain Relatedness by Rep-PCR

**Figure 2:**
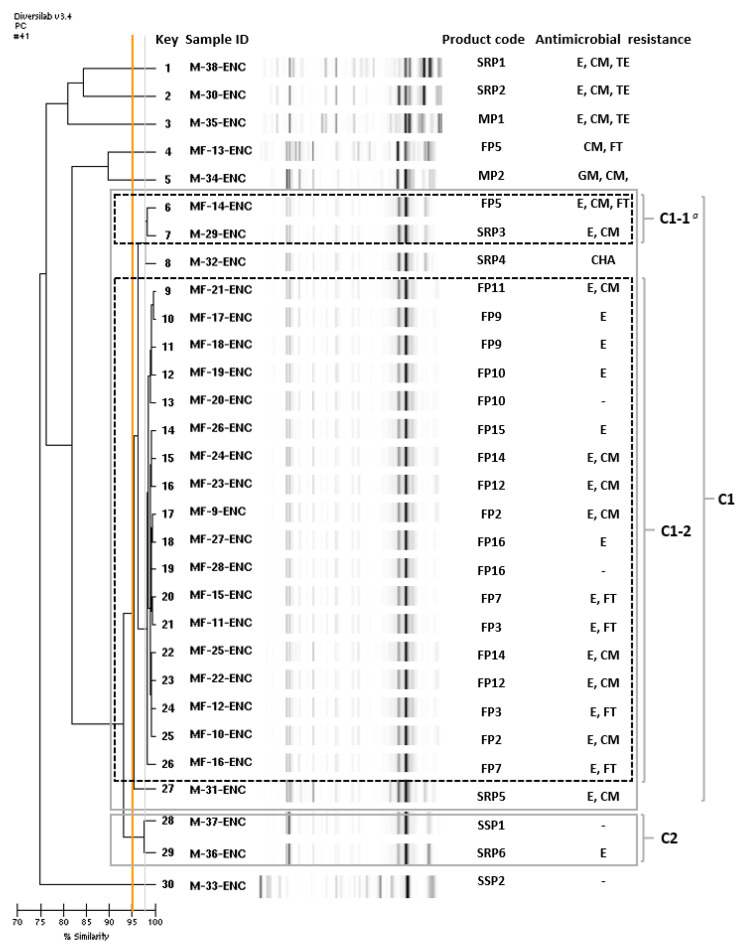
Molecular typing of *Enterococcus faecium* isolates obtained from the commercial processed pork meat products in Korea. The dendrogram shows the molecular typing results obtained by rep-PCR with the Diversilab method. The total number of isolates analyzed in this study was 30. Clusters with Diversilab similarity ≥95% are marked with a grey rectangle and numbered (C1 and C2). Clusters with a similarity ≥98% are marked with a black dotted line and numbered C1-1 and C1-2. The abbreviation in the product code represents MP (minced pork meat), SRP (ready-to-cook pork meat products seasoned with red pepper paste), SSP (ready-to-cook pork meat products seasoned with soy sauce) and FP (frozen meatball-type processed pork meat products). Antimicrobial resistances were observed for Erythromycin (E), Clindamycin (CM), Tetracycline (TE), Nitrofurantoin (FT), Chloramphenicol (CHA) and Gentamicin (GM). ^a^ Cluster number.

To identify the strain relatedness of *E. faecium* isolates obtained from different types of processed pork meat products, molecular typing was performed with Diversilab, an automated rep-PCR method. Initial analysis of rep-PCR dendrograms showed two clusters (C1 and C2) with a similarity ≥ 95% and 6 singleton isolates in Figure 2. In the cluster C1, distinctive sub-clusters with a similarity ≥ 98% were detected as C1-1 and C1-2. Surprisingly, 18 out of the 20 *E. faecium* isolates originated from FP were clustered as C1-2 and most of the *E. faecium* isolates clustered as C1-2 were resistant to erythromycin (Figure 2). Rep-PCR typing using Diversilab has value as a semi-automated, very quick, certified genotyping method using a centralized and standardized analysis software. Thus, it can be implemented in a routine diagnostic laboratory workflow [31]. Various molecular typing methods are used for the surveillance of foodborne pathogens and outbreak investigations, differing widely in information content and discriminatory power. Presently, the focus is shifting to whole genome sequencing (WGS) as an analytical tool [32]. In this study, we only provided the rep-PCR-based molecular typing *E. faecium*, but additional molecular or phenotypic analysis including Pulsed field gel electrophoresis (PFGE), MLST and WGS is recommended to further elucidate the rep-PCR typing result.

The high strain relatedness of *E. faecium* isolated from FP products might be explained by: (1) *E. faecium* isolates might be contaminated in the common ingredients used in the varieties of FP products analyzed in this study, or (2) *E. faecium* isolates might be cross-contaminated from the processing environment including water of the manufacturing company. To prove the first assumption, i.e., *E. faecium* isolates might originate from ingredients of FP products, sources and content of the meat used as main ingredients together with information on the product name and manufacturing company are compared.

As in Table 4, all the FP products contained domestically produced pork meat except for one sample (Key No. 21), but contents of the pork meats were different (33.3~75.2%) according to the product type. Other types of processed pork meat products (M, SRP, SS) also contained domestically produced pork meat.

Chicken meat is also frequently used as an ingredient of FP products: 16 out of 20 samples of FP products contained chicken meat as ingredients. However, *E. faecium* strains originated from FP products containing pork meat only (Key No. 10, 11, 18, 19) showed high similarity (≥98%) with *E. faecium* isolated from FP products containing chicken and pork meat (Key No. 4, 6, 9, 12–17, 20–26) as shown in Figure 2 and Table 4. Therefore, *E. faecium* clustered as C1-2 might originate from ingredients other than pork and chicken meat. Most of the FP products contain seasoning and vegetables such as green onion but no information on the content of the vegetables or details of seasoning was provided in the food label of FP products. To identify the exact sources of *E. faecium* contamination, more detailed information on the minor ingredients, for example, seasoning, are needed, and prevalence of enterococci in the ingredients and molecular typing of the enterococci isolates should be analyzed.

**Figure 3:**
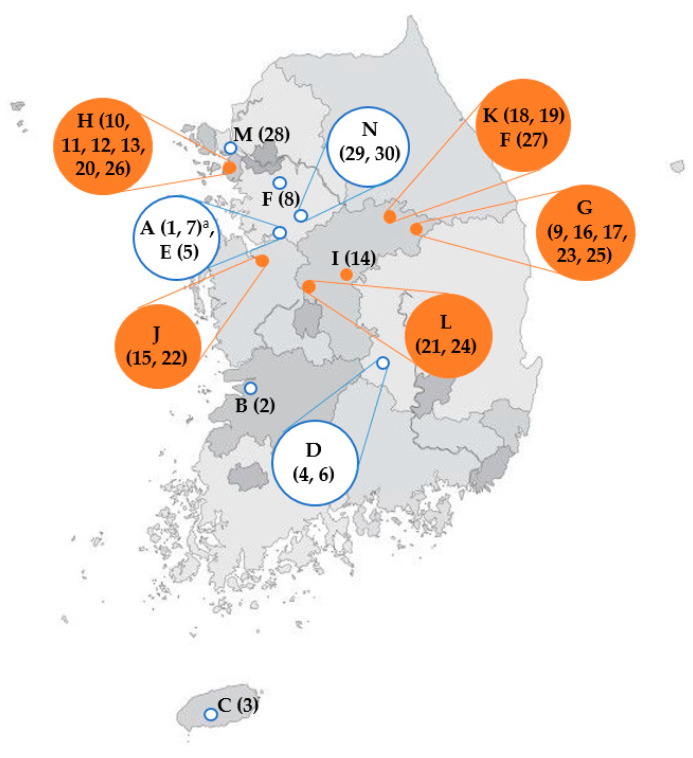
Location of processing company for pork meat products analyzed for *E. faecium* contaminations. Orange closed circles and blue open circles represent the geographical position of processing company produced samples analyzed in this study. Orange closed circles indicate the geographical position of processing company produced samples contaminated with closely related *E. faecium* with a similarity ≥98%, which were analyzed by automated rep-PCR in Figure 2. ^a^ A (1, 7): Alphabet indicates the processing company. Numbers in parentheses depict the key number in the dendrogram of rep-PCR and Table 4.

For the second assumption, that the *E. faecium* isolates might originate from processing environments or water, the best way to prove the hypothesis is to analyze the occurrence of *E. faecium* in the processing environments including water [33]. However, we were not able to access the processing environments of the manufacturing companies of FP products to monitor the occurrence of enterococci, unfortunately. Instead, manufacturing companies of FP products and locations of each company were analyzed as an indirect approach. As shown in Table 4 and Figure 3, FP products analyzed in this study were manufactured at seven different companies (Company D, G, H, I, J, K and L) located in scattered areas of Korea. The closely related *E. faecium* isolates originated from FP products manufactured at seven companies with different geographical locations, suggesting very low possibilities of cross-contamination of *E. faecium* from processing environments.

In the food supply chain, the pathogenic potential of enterococci is of concern due to their ability to harbor and transfer virulence and antimicrobial resistance to other pathogens based on horizontal gene transfer. Although *E. faecium* tested in this study have low MDR, 85% of the *E. faecium* isolated from FP showed erythromycin resistance (Figure 2), which is categorized as a critically important antimicrobial for human medicine [22]. Because spread of antimicrobial resistance, especially community-acquired antimicrobial resistance, is increasing and food can be a good source of contamination for humans, early detection of the transmission of antimicrobial-resistant enterococci in the food chain is important to control antimicrobial resistance due to food and food environments [34].

## 4. Conclusions

In conclusion, contamination levels of *Enterococcus* spp. were analyzed in processed pork meat products (*n* = 124) commercially available in Korea. We found that contamination in the MP is the highest followed by SRP and SSP. For the 30 *E. faecium* isolates, antimicrobial resistance was analyzed against 14 antimicrobials belonging to 12 antimicrobial classes. The highest incidence of antimicrobial-resistant *E. faecium* was observed for erythromycin (80% resistance) followed by clindamycin (50%) and nitrofurantoin (20%). No vancomycin-resistant enterococci were observed. Rep-PCR showed distinctive clusters with a similarity ≥98% consisting of 18 *E. faecium* isolates from FP manufactured in seven companies, suggesting that *E. faecium* isolates obtained from FP may be closely related. The results from Rep-PCR would reflect the real relatedness of *E. faecium* isolates, but additional molecular or phenotypic analysis including PFGE, MLST and WGS is recommended to further elucidate the rep-PCR typing result. High prevalence of erythromycin-resistant *E. faecium* among the clustered enterococci suggested that antimicrobial-resistant enterococci might be transmitted via food and cause food safety and public health concerns. Information on the contamination and antimicrobial resistance patterns combined with molecular typing analyzed in this study can be useful to derive risk management options, preventing the spread of antimicrobial-resistant enterococci in food.

## Figures and Tables

**Table 1:** Prevalence of *Enterococcus faecium* and *Enterococcus faecalis* in commercially available processed pork meat products.

Conditions at Retail Market	Type of Products	Isolated *Enterococcus* spp.	Number of Positive Samples/Number of Total Samples (%)	
			*E. faecium*	*E. faecalis*
Refrigerated	Minced	*E. avium*, *E. faecalis*, *E. faecium*, *E. raffinosus*	1/40 (2.5%)	12/40 (30.0%)
	Seasoned with red pepper paste	*E. avium*, *E. casseliflavus*, *E. faecalis*, *E. faecium*, *E. gallinarum*, *E. raffinosus*	5/34 (14.7%)	5/34 (14.7%)
	Seasoned with soy sauce	*E. avium*, *E. durans*, *E. faecalis*, *E. faecium*, *E. raffinosus*	2/34 (5.9%)	3/34 (8.8%)
	Subtotal	*E. avium*, *E. casseliflavus*, *E. durans*, *E. faecalis*, *E. faecium*, *E. gallinarum*, *E. raffinosus*	8/108 (7.4%)	20/108 (18.5%)
Frozen	Meatball-type products	*E. casseliflavus*, *E. faecalis*, *E. faecium*	11/16 (68.8%)	5/16 (31.3%)
Total		*E. avium*, *E. casseliflavus*, *E. durans*, *E. faecalis*, *E. faecium*, *E. gallinarum*, *E. raffinosus*	19/124 (15.3%)	25/124 (20.2%)

**Table 2:** Antimicrobial resistance of *Enterococcus faecium* and *Enterococcus faecalis* against 14 antibiotics.

Antimicrobial Class	Antimicrobials	No. of *E. faecium* Isolates (%)			No. of *E. faecalis* Isolates (%)		
		R ^a^	I	S	R	I	S
Quinolones	Ciprofloxacin	0 (0.0)	4 (13.3)	26 (86.6)	0 (0.0) ^b^	4 (11.1)	32 (88.9)
Macrolides and ketolides	Erythromycin	24 (80.0)	5(16.7)	1 (3.3)	4 (11.1) ^b^	14 (38.9)	18 (50.0)
Oxazolidinones	Linezolid	0 (0.0)	0 (0.0)	30 (100.0)	0 (0.0) ^b^	1 (2.8)	35 (97.2)
Nitrofurantoins	Nitrofurantoin	6 (20.0)	20 (66.6)	4 (13.3)	1 (2.8) ^b^	8 (22.2)	27 (75.0)
Glycopeptides	Teicoplanin	0 (0.0)	0 (0.0)	30 (100.0)	0 (0.0) ^b^	0 (0.0)	36 (100.0)
Glycopeptides	Vancomycin	0 (0.0)	0 (0.0)	30 (100.0)	0 (0.0) ^b^	0 (0.0)	36 (100.0)
Tetracyclines	Tetracycline	3 (10.0)	0 (0.0)	27 (90.0)	21 (58.3) ^b^	0 (0.0)	15 (41.7)
Glycylcyclines	Tigecycline	0 (0.0)	0 (0.0)	30 (100.0)	0 (0.0) ^b^	0 (0.0)	36 (100.0)
Penicillins	Amoxycillin/clavulanic acid	0 (0.0)	0 (0.0)	30 (100.0)	0 (0.0) ^b^	0 (0.0)	36 (100.0)
Penicillin	Aampicillin	0 (0.0)	0 (0.0)	30 (100.0)	0 (0.0) ^b^	0 (0.0)	36 (100.0)
Amphenicol	Chloramphenicol	1 (3.3)	1 (3.3)	28 (93.3)	0 (0.0) ^b^	0 (0.0)	36 (100.0)
Streptogramins	Quinupristin/dalfopristin	0 (0.0)	0 (0.0)	30 (100.0)	25 (69.4)	6 (16.7)	5 (13.9)
Lincosamides	Clindamycin	15 (50.0)	8 (26.6)	7 (23.3)	32 (88.9)	0 (0.0)	4 (11.1)
Aminoglycosides	Gentamicin	1 (3.3)	0 (0.0)	29 (96.7)	0 (0.0)	0 (0.0)	36 (100.0)

^a^ R, resistant; I, intermediate resistance; and S, sensitive to the antimicrobials. ^b^ Antimicrobial resistance of *E. faecalis* against ciprofloxacin, erythromycin, linezolid, nitrofurantoin, teicoplanin, vancomycin, tetracycline, tigecycline, amoxycillin/clavulanic acid, ampicillin and chloramphenicol were adopted from our previous study for comparison with antimicrobial resistance of *E. faecium* [23]. Antimicrobial resistance of the 36 *E. faecalis* against quinupristin/dalfopristin, clindamycin and gentamicin were analyzed in the present study.

**Table 3:** Multidrug resistance of *Enterococcus faecium* isolated from processed pork meat products.

Resistant Antimicrobials	Number of Strains (%) for Different Types of Pork Meat Products				
	MP *^b^*	SRP	SSP	FP	Total
None	0 (0.0)	0 (0.0)	2 (6.7)	2 (6.7)	4 (13.3)
CHA *^a^*	1 (3.3)	0 (0.0)	0 (0.0)	0 (0.0)	1 (3.3)
GM	0 (0.0)	0 (0.0)	0 (0.0)	0 (0.0)	0 (0.0)
E	0 (0.0)	1 (3.3)	0 (0.0)	5 (16.7)	6 (20.0)
FT	0 (0.0)	0 (0.0)	0 (0.0)	0 (0.0)	0 (0.0)
GM, CM	1 (3.3)	0 (0.0)	0 (0.0)	0 (0.0)	1 (3.3)
E, CM	0 (0.0)	2 (6.7)	0 (0.0)	7 (23.3)	9 (30.0)
E, FT	0 (0.0)	0 (0.0)	0 (0.0)	4 (13.3)	4 (13.3)
CM, FT	0 (0.0)	0 (0.0)	0 (0.0)	1 (3.3)	1 (3.3)
E, TE	0 (0.0)	0 (0.0)	0 (0.0)	0 (0.0)	0 (00.0)
E, TE, CM	0 (0.0)	3 (10.0)	0 (0.0)	0 (0.0)	3 (10.0)
E, CM, FT	0 (0.0)	0 (0.0)	0 (0.0)	1 (3.3)	1 (3.3)

Antimicrobial resistance of 30 *E. faecium* isolates were tested against 14 antimicrobials, including gentamicin, ciprofloxacin, tetracycline, ampicillin, chloramphenicol, erythromycin, amoxycillin/clavulanic acid, clindamycin, quinupristin/dalfopristin, linezolid, teicoplanin, vancomycin, tigecycline and nitrofurantoin. *^a^* CHA: chloramphenicol, GM: gentamicin, E, erythromycin; FT, nitrofurantoin; TE, tetracycline. CM: clindamycin. *^b^* Each abbreviation represents the type of product as follows: MP: minced pork meat, SRP: ready-to-cook pork meat seasoned with red pepper paste, SSP: ready-to-cook pork meat seasoned with soy sauce, FP: frozen meatball-type processed pork meat products.

**Table 4:** Information on the pork meat products analyzed for *E. faecium.*

Key No. *^a^*	Product Code	Manufacturing Company	Main Ingredients
1	SRP1	A *^b^*	Pork
2	SRP2	B	Pork
3	MP1	C	Pork
4	FP5	D	Pork (66.03%), Chicken (4.4%)
5	MP2	E	Pork 100%
6	FP5	D	Pork (66.03%), Chicken (4.4%)
7	SRP3	A	Pork meat
8	SRP4	F	Pork meat
9	FP11	G	Pork (20.56%), chicken (38.16%)
10	FP9	H	Pork (75.2%)
11	FP9	H	Pork (75.2%)
12	FP10	H	Pork (60.94%), chicken
13	FP10	H	Pork (60.94%), chicken
14	FP15	I	Pork (30.6%), chicken (22.95%), beef (7.65%)
15	FP14	J	Pork (29.18%), chicken (20.84%)
16	FP12	G	Pork (50.74%), Chicken (19.73%)
17	FP2	G	Pork (62.22%), Chicken (6.89%)
18	FP16	K	Pork (78.43%)
19	FP16	K	Pork (78.43%)
20	FP7	H	Pork (33.37%), chicken (12.05%)
21	FP3	L	Pork (domestic 59.2%, imported 14.8%)
22	FP14	J	Pork (29.18%), chicken (20.84%)
23	FP12	G	Pork (50.74%), Chicken (19.73%)
24	FP3	L	Pork (domestic 59.2%, imported 14.8%)
25	FP2	G	Pork (62.22%), Chicken (6.89%)
26	FP7	H	Pork (33.37%), chicken (12.05%)
27	SRP5	F	Pork
28	SSP1	M	Pork (70%)
29	SRP6	N	Pork (70%)
30	SSP2	N	Pork (70%)

*^a^* Key number are adopted from rep-PCR data in Figure 2. *^b^* Locations of the manufacturing company were presented in Figure 3. All the pork meat used as the main ingredient was domestically grown, except for FP3 (key number 21).
